# Removing Critical Gaps in Chemical Test Methods by Developing New Assays for the Identification of Thyroid Hormone System-Disrupting Chemicals—The ATHENA Project

**DOI:** 10.3390/ijms21093123

**Published:** 2020-04-28

**Authors:** Andreas Kortenkamp, Marta Axelstad, Asma H. Baig, Åke Bergman, Carl-Gustaf Bornehag, Peter Cenijn, Sofie Christiansen, Barbara Demeneix, Arash Derakhshan, Jean-Baptiste Fini, Caroline Frädrich, Timo Hamers, Lina Hellwig, Josef Köhrle, Tim I.M. Korevaar, Johan Lindberg, Olwenn Martin, Marcel E. Meima, Philipp Mergenthaler, Nikolai Nikolov, David Du Pasquier, Robin P. Peeters, Bjorn Platzack, Louise Ramhøj, Sylvie Remaud, Kostja Renko, Martin Scholze, Harald Stachelscheid, Terje Svingen, Fabian Wagenaars, Eva Bay Wedebye, R. Thomas Zoeller

**Affiliations:** 1 Institute of Environment, Health and Societies, Brunel University London, Uxbridge UB8 3PH, UK; 2 National Food Institute, Technical University of Denmark, DK-2800 Kgs. Lyngby, Denmark; 3 School of Science and Technology, Orebro University, SE-701 82 Orebro, Sweden; 4 Department of Health Sciences, Karlstad University, SE-651 88 Karlstad, Sweden; 5 Department of Environment and Health, Vrije Universiteit Amsterdam, VUA, 1081 HV Amsterdam, The Netherlands; 6 Unité PhyMA Laboratory, Adaptation du Vivant, Muséum national d’Histoire naturelle, Centre National de la Recherche Scientifique CNRS 7, rue Cuvier, F-75005 Paris, France; 7 Department of Internal Medicine, Academic Center for Thyroid Diseases, Erasmus Medical Centre, 3000 CA Rotterdam, The Netherlands; 8 Department of Experimental Endocrinology, Charitė - Universitätsmedizin Berlin, D-13353 Berlin, Germany; 9 Dept. of Experimental Neurology, Dept. of Neurology, Center for Stroke Research Berlin, Charité – Universitätsmedizin Berlin, D-10117 Berlin, Germany; 10 Charité-BIH Centrum Therapy and Research, BIH Stem Cell Core Facility, Charité – Universitätsmedizin Berlin, D-13353 Berlin, Germany; 11 Department of C4hemical Process and Pharmaceutical Development, Research Institutes Sweden, RISE, SE-151 36 Sodertalje, Sweden; 12 Berlin Institute of Health, D-10178 Berlin, Germany; 13 Laboratoire Watchfrog, F-91000 Evry, France

**Keywords:** endocrine disruptors, thyroid hormone system, brain development, test method development, test method validation, risk assessment

## Abstract

The test methods that currently exist for the identification of thyroid hormone system-disrupting chemicals are woefully inadequate. There are currently no internationally validated in vitro assays, and test methods that can capture the consequences of diminished or enhanced thyroid hormone action on the developing brain are missing entirely. These gaps put the public at risk and risk assessors in a difficult position. Decisions about the status of chemicals as thyroid hormone system disruptors currently are based on inadequate toxicity data. The ATHENA project (Assays for the identification of Thyroid Hormone axis-disrupting chemicals: Elaborating Novel Assessment strategies) has been conceived to address these gaps. The project will develop new test methods for the disruption of thyroid hormone transport across biological barriers such as the blood–brain and blood–placenta barriers. It will also devise methods for the disruption of the downstream effects on the brain. ATHENA will deliver a testing strategy based on those elements of the thyroid hormone system that, when disrupted, could have the greatest impact on diminished or enhanced thyroid hormone action and therefore should be targeted through effective testing. To further enhance the impact of the ATHENA test method developments, the project will develop concepts for better international collaboration and development in the area of thyroid hormone system disruptor identification and regulation.

## 1. Introduction

The development of internationally validated test methods for chemicals that disrupt the thyroid hormone system has not kept pace with the scientific advances in the field of thyroid hormone (TH) endocrinology and physiology. As a result, we are not protected against “surprises” from chemicals that suddenly reveal their potential as TH system disruptors with adverse consequences for brain development in foetal and neonatal life.

A major weakness of the currently validated test methods is the lack of assays specific to the impairment of brain development. This puts risk assessors in a difficult position: unable to base their evaluations on relevant brain-related endpoints, the danger of developmental neurotoxicity must be inferred from data related to histopathological changes in the thyroid gland or TH serum concentrations, which are taken as evidence of the disruption of the thyroid hormone system. Whether or not this also leads to developmental neurotoxicity is often unclear, especially in cases when the concentrations of circulating thyroid hormones are perturbed, but without histological changes in the thyroid [1].

The ATHENA consortium, funded by the European Union (EU) Horizon 2020 programme, aims to close this disturbing gap by mobilizing the scientific progress made in recent years regarding our understanding of the TH system. We focus on TH system disruption that arises from interference with TH transport processes to the foetus, from disturbances of the cellular uptake and intra-cellular deiodination of TH and from disruptions of the local control of the receptor-mediated action of TH in target cells. We will establish new toxicity endpoints that will allow us to capture the downstream effects of TH system disruption on the developing brain in foetal and post-natal life.

**Figure 1:**
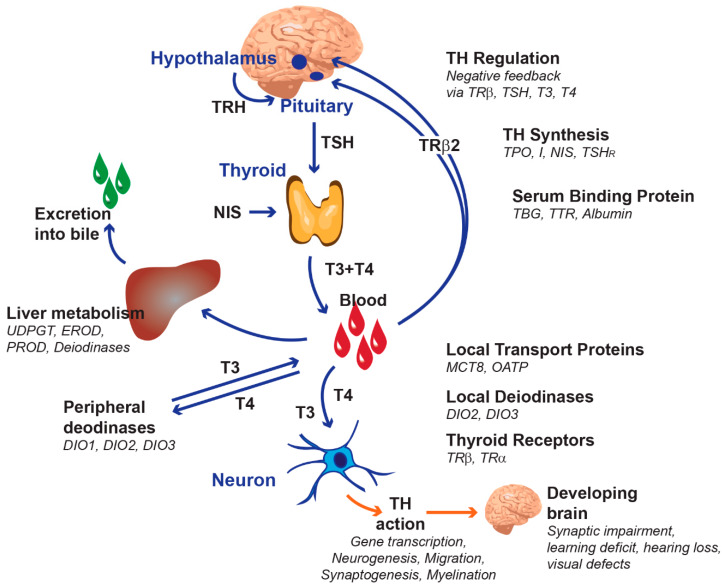
The thyroid hormone system and its regulation. (adapted and modified from Gilbert et al. (2012) [2]). Abbreviations are listed at the end of the paper.

THs play essential roles in growth and development by controlling cell differentiation, migration and homeostasis. Hormone synthesis is regulated by the hypothalamic–pituitary–thyroid axis. Effective tissue levels of THs are controlled through multiple layers of regulation including deiodinations in peripheral tissues, specific cell membrane transporters, binding to distributor proteins, the removal of TH by sulfation or glucuronidation in the liver, or the modulation of iodide uptake into the thyroid (Figure 1) [2]. All these mechanisms influence the availability of THs in the receptors inside target cells. This complex regulatory system can be disrupted at multiple levels—for example, by chemicals that suppress TH synthesis, interfere with the uptake of iodide into the thyroid gland, block the transfer of THs across physiological barriers or stimulate the clearance of THs in the liver [2].

In humans, both too low and too high serum TH availability are associated with negative health impacts [3]. Non-optimal thyroid hormone availability during pregnancy has been associated not only with suboptimal child neurocognitive and brain morphological outcomes, but also pre-eclampsia, intrauterine growth restriction and preterm birth. Outside of pregnancy, non-optimal TH availability is associated with a higher risk of cardiovascular disease, cardiac arrhythmias, osteoporosis and the disturbance of lipid metabolism. For this reason, the ATHENA project will focus on pre-receptor TH regulatory mechanisms that lead to TH system disruption. We will develop test methods that can capture adverse outcomes on the developing brain. The project will be completed at the end of 2023.

In this article, we describe the concept and approaches of the ATHENA project [4], give an overview of some first achievements and discuss the relevance of ATHENA in the context of the international regulatory landscape. ATHENA is part of the European Cluster to Improve Identification of Endocrine Disruptors (EURION, https://eurion-cluster.org), which is composed of eight EU-funded projects which collaborate on test method development.

## 2. Results

### 2.1. Terminology, Concept and Rationale

There are ambiguities in the naming of chemicals that can disrupt the functions of the TH system. Frequently, these are referred to as “thyroid disruptors”, but this is sometimes taken to apply only to chemicals that disrupt the thyroid gland directly. Interference with other aspects of the TH system, such as effects on serum distributor proteins, cell membrane transporters, intracellular deiodinases (DIO) or liver-conjugating enzyme systems for the elimination of TH, to name a few, are then not taken into account and referred to as merely “indirect” effects. However, disturbances of any of these aspects can also lead to adverse effects. To express this insight, we avoid the term “thyroid disruptor” throughout this article. Instead, we use the term “TH system disruptors” to refer to substances with the ability of disrupting any aspect of the TH system. Accordingly, the process itself is called “TH system disruption”.

The ATHENA concept for advancing improved and novel test methods for the detection of TH system disruptors provides enhancements on several levels:

- The development of quantitative structure–activity relationships (QSARs) for molecular initiating events leading to diminished TH action, to allow for the improved screening of candidate TH system disruptors;
- The scaling up of in vitro methods for the inhibition of deiodinases, dehalogenases and cell membrane TH transporters to a high throughput format;
- The development of new assays to capture the disruption of the delivery of THs across physiological barriers (cell membrane transporters in the blood–brain barrier, the blood–cerebrospinal fluid barrier, and the placenta);
- The development of new assays based on human pluripotent stem cell-derived brain cells and brain organoids to complement and, to a certain extent, replace current animal models;
- The identification of endpoints reflective of brain morphology and markers of brain development for possible inclusion in existing Organisation for Economic Co-operation and Development (OECD) test guidelines;
- The integration of new and existing test methods into a coherent testing strategy;
- The validation of the human relevance of new test methods.

The realisation of some of these aspects will require basic research, while others can build on existing assays that need further refinement to reach the pre-validation stage. There is an urgent need to integrate test methods by elaborating coherent testing strategies that can support effective hazard characterisations and regulatory decisions. These testing strategies will specify sequential deployments of tests to comprehensively capture all aspects of TH system disruption. There is also the need for international harmonisation of hazard characterisations for TH system disruptors.

### 2.2. Regulatory and Societal Needs for New Test Methods for Thyroid Hormone System Disruption

The need to identify the TH system-disrupting potential of chemicals derives from EU legal requirements for the protection against endocrine-disrupting chemicals expressed in various EU chemicals regulations (e.g., Biocidal Products Regulation (EU) No 528/2012, Plant Protection Products Regulation (European Commission) No 1107/2009, Regulation on Registration, Evaluation, Authorisation and Restriction of Chemicals (REACH; European Commission 1907/2006)). It is widely acknowledged that the internationally validated and accepted OECD test methods for endocrine disruptors are inadequate for the identification of TH system-disrupting chemicals [1]. Therefore, the legal mandate for protection against endocrine disruptors and TH system disruptors cannot currently be fully realised.

Although in vitro methods that capture various aspects of TH synthesis, transport and metabolism [5] have been available for a considerable period, none of these methods are validated internationally and therefore are not used for regulatory testing. As a result, there is currently not a single in vitro method for TH system disruptors implemented in Level 2 of the OECD Conceptual Framework for endocrine disruptor testing [1]. Similarly, Level 3 (in vivo assays providing data about selected endocrine mechanisms) does not specify any thyroid-related endpoints. Moreover, the Level 4 test guideline (TG) 407 (repeated dose 28-day study, adult animals) only includes thyroid gland histopathology, with optional TH measurements. An update of TG 408 (repeated dose 90-day oral toxicity) includes mandatory TH measurements. In recent years, some Level 4 tests (reproductive toxicity screening and developmental toxicity) have been amended to include TH measurements in some of the animals. At Level 5 of the OECD Conceptual Framework, only the Extended One-Generation Reproductive Toxicity Study (EOGRTS, TG 443) includes mandatory TH measurements, but downstream effects of diminished TH action on the brain are insufficiently covered and poorly developed. The currently available neuro-behavioural tests used to capture this kind of developmental neurotoxicity are rarely included in regulatory testing studies. As a result, most chemicals have not been tested for potential developmental neurotoxicity. In any case, the available neuro-behavioural tests are normally not sufficiently sensitive for the reliable detection of TH system disruptors.

The lack of adequate test methods severely constrains the evaluation of chemicals as TH system disruptors. The absence of reliable downstream effect markers for disturbance of brain development and the deficiencies of in vitro methods mean that chemicals have to be assessed mainly on the basis of their capability of inducing histopathological changes in the thyroid and in terms of their ability to alter TH serum levels. However, modulations of hormone levels alone give an inadequate reflection of a chemical’s ability to disrupt the TH system. Furthermore, the thyroid is not the only target organ of the TH system. The result is that the TH system-disrupting properties of many chemicals are insufficiently identified or even entirely overlooked, especially in relation to the disruption of foetal brain development.

Furthermore, coherent testing strategies for the identification of TH system-disrupting chemicals are missing altogether. It can be anticipated that exposures that limit the synthesis of THs by restricting the uptake of iodide into the thyroid gland, or by blocking hormone-synthesising enzymes, will have a strong impact on TH action. It remains to be seen whether a testing strategy should begin with in vitro assays for uptake inhibition, or the inhibition of hormone synthesis, or whether other molecular initiating events should also be considered. It is further necessary to elaborate which tests should be deployed in the case of positive outcomes of initial in vitro tests, and when the scene might be set to proceed to testing with cost-intensive multi-generation in vivo mammalian assays.

### 2.3. Overall Approach and Methodology

The ATHENA project is organised into four project domains:

- Domain 1: Mobilising basic research for the development of novel assays for TH system disruption;
- Domain 2: The development of test methods towards assay pre-validation and ring-testing;
- Domain 3: Testing strategies and testing in a multi-causal context;
- Domain 4: Enhanced international collaboration and development of international strategies for TH system disruptor identification and regulation.

#### 2.3.1. Domain 1: Mobilising Basic Research for the Development of Novel TH System Disruption Assays

In this domain, we conduct basic research that can orient and underpin the development of test methods in domain 2 of the project, in terms of aspects of the TH system that need to be addressed by test methods, the selection of test chemicals, as well as features of human physiology not readily re-capitulated in experimental studies and in vitro and in vivo test methods.

We are leveraging human epidemiological data to guide the decision-making process for test method development and test compound selection. We are interrogating data from several epidemiological cohorts to take stock of the known associations of chemical exposures with adverse health outcomes related to maternal thyroid function. This will provide a basis for the selection of test chemicals and components of the TH system that should be the initial targets when setting up assay development. We have identified the associations of several endocrine-disrupting chemicals with disruptions of maternal thyroid function using epidemiological data from the Swedish Environmental Longitudinal, Mother and child, Asthma and allergy (SELMA) cohort and the Generation R study [6]. To complement our work, we will also use experimental data with newly discovered TH system-disrupting chemicals to probe possible exposure effect associations in human epidemiological data. We have successfully used human epidemiological data to elucidate TH system-disrupting effects that are specific to human pregnancy physiology and which cannot be examined in experimental systems [6].

**Figure 2:**
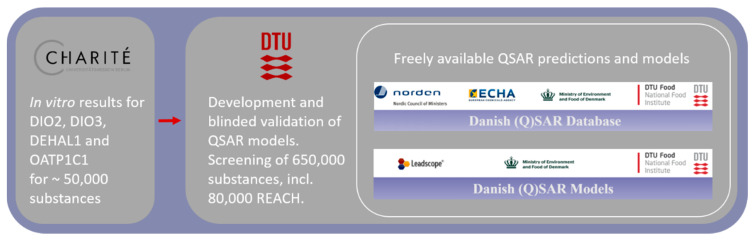
Relationship between quantitative structure–activity relationship (QSAR) development and results from high throughput assays in domain 2 of ATHENA.

We are screening for candidate TH system disruptors by developing advanced QSAR models. This work builds up valuable criteria for test chemical selection from a molecular/mechanistic perspective. We are developing new QSARs for the inhibition of deiodinases, dehalogenase and TH membrane transporters in order to screen REACH chemicals, pesticides, biocides and pharmaceuticals for their potential to disrupt components of the TH system. The results from the high throughput assays to be developed and refined in domain 2 of ATHENA (see below) will produce comprehensive datasets, which will be used to design and refine complex and well-trained QSAR algorithms that can characterise chemicals as potential TH system disruptors (Figure 2).

**Figure 3:**
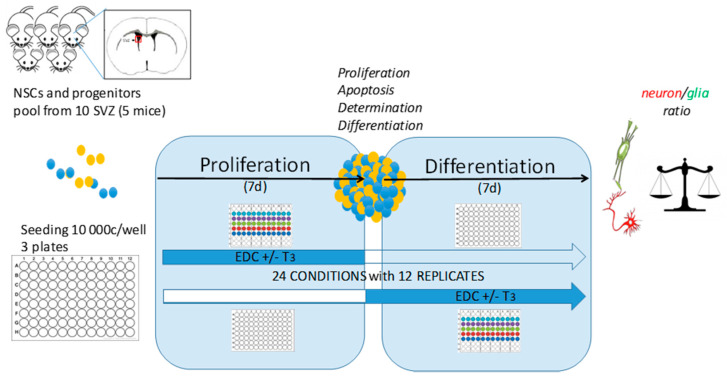
Protocol for developing neurospheres from two-month-old mice. Neurospheres prepared from the subventricular zone (SVZ) of five mice are obtained after 7 days of proliferation in the presence of growth factors. Without growth factors, neurosphere dissociated cells are allowed to differentiate into neuronal or glial cells. Thyroid hormones tightly influence this balance by promoting the neuronal fate at the expense of the glial fate. Note that thyroid hormone (TH) antagonists induce the opposite phenotype.

We are developing in vitro and ex vivo three-dimensional (3-D) models to determine the disruption of neural cell fate decisions by using two models, (i) human brain organoids and (ii) neurospheres established from the subventricular zone of postnatal and adult mice (Figure 3). We have also derived brain organoids that were generated from human-induced pluripotent stem cells [7]. These systems will help us to establish how TH system-disrupting chemicals can interfere with brain development, neural stem cell differentiation and key processes during foetal brain development. These highly promising novel approaches mimic the complex three-dimensional intercellular communication between different types of brain cells (e.g., neurons, oligodendrocytes, astrocytes). These assays will undergo technical refinement and optimisation in an iterative process of broad applications, the goal being to reduce, refine and replace animal experiments (3R) and to increase the throughput capacity of in vitro and ex vivo test methods. This research will provide the basis for novel in vitro/ex vivo test methods and links directly with the in vivo studies of thyroid hormone downstream effects on the developing brain undertaken in domain 2. Together with these in vivo test methods, the work with human brain organoids and mouse neurospheres will provide a compelling platform for examining the impact of the disruption of TH transport into brain cells and the impact of the interference with local TH activations or inactivations on cell fate decisions.

#### 2.3.2. Domain 2: Development of Test Methods towards Assay Pre-Validation and Ring-Testing

In this domain, we are conducting the research and method optimisations required to take existing TH system disruptor assays from the pilot and research stages to the test method pre-validation stage. We are carrying out thorough assessments in terms of assay reliability, sensitivity, efficiency and technical feasibility at a high throughput screening (HTS) level, based on rigorous statistical and biometrical analyses. This work incorporates all aspects important to the international validation process in the OECD Test Guideline Framework. The work in domain 2 probes the TH system with a focus on TH metabolism (deiodination, dehalogenation), transport processes across physiological barriers and downstream effects on the developing foetal brain. Our work complements the efforts currently undertaken in the Network of Laboratories for the Validation of Alternative Methods (NETVAL) process coordinated by the European Commission’s Joint Research Centre.

We are developing high throughput screening (HTS) for specific inhibitors of iodothyronine deiodinase 2 & 3, iodotyrosine dehalogenase 1 (IYD) and TH transporter organic anion transporter 1c1 (OATP1C1) to generate QSAR training sets and reference compound libraries. This builds on established assay protocols, developed by ATHENA partners. These protocols utilise a convenient colorimetric, non-radioactive method for the detection of iodine based on the Sandell–Kolthoff reaction. A panel of recently developed assays [8] is scaled up to a new level of throughput and superior quality. These will be optimised for a high throughput format (384-well plate) and semi-automated handling. We will deliver a common assay platform in which distinct DIOs, IYDs, and recently discovered cell membrane TH transporters can be tested. A key benefit of this work will be its transferable and high-quality protocols, which will be ready for validation and adoption by user labs. We will perform the screening of large compound libraries, consisting of a diverse set of orphan molecules, including pre-selected REACH chemicals. This screening will deliver training sets for QSAR development and refinement in domain 1. Conversely, we will verify experimentally QSAR-predicted active REACH chemicals. The screening effort will also generate a valuable library of specific and selective inhibitors of deiodinases, dehalogenases and membrane transporters. We will use this library in other in vivo and ex vivo assays to establish the outcomes and biomarkers that result from these specific modes of action of disrupting the TH system.

We are analysing the disruption of TH transport across physiological barriers and are scaling up existing cell models genetically engineered to overexpress the established transporters monocarboxylate transporter (MCT 8/10) and OATP1C1 for high throughput assays. We utilise non-radioactive iodine detection methods (Sandell–Kolthoff reaction). The aim is to deliver HTS protocols ready for pre-validation via the extension and refinement of existing protocols [9]. We will conduct basic research on identifying the main TH transporters in the placenta, the blood–brain barrier and the blood–cerebrospinal fluid barrier by using established polarised cell models in trans-well cultures and by using perfused placenta models. Newly identified, novel transporters are overexpressed in established cell lines and the resulting genetically engineered cell lines are further developed into high throughput assays for the identification of chemicals capable of disrupting TH transporters. This work will be complemented and expanded by studying the role of the distributor protein transthyretin (TTR) in TH transport across the polarised cell models, representing the placenta and the blood–cerebrospinal fluid barrier. TTR is the main TH binding protein in the cerebrospinal fluid and is directionally secreted from choroid plexus epithelial cells, which form these barriers, into the cerebrospinal fluid. Similarly, TTR is directionally secreted by placental cells into the foetal environment, where it represents the relevant TH-binding protein. Recently, we have assessed the impact of mixtures of chemicals on displacing THs from TTR in serum [10].

**Figure 4:**
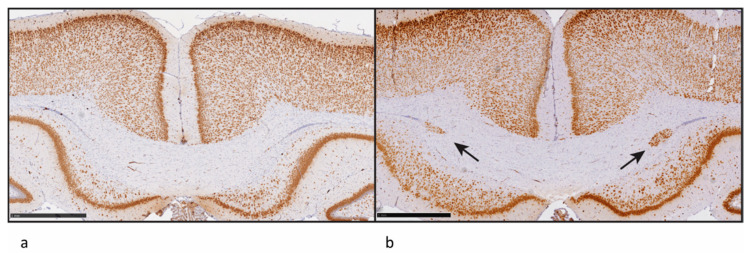
Heterotopia—misplaced cells in the corpus callosum of rats. Panel (**a**), left, shows the appearance of the corpus callosum in a control rat. Panel (**b**), right, shows heterotopia in the corpus callosum of a rat exposed in foetal life and postnatally to propylthiouracil (maternal dose: 2.5 mg/kg d). The arrows point to the misplaced cells. Scale bars: 1 cm.

We have explored the feasibility of using endpoints reflective of disruptions of differentiation, maturation and migration of brain cells during the development of in vivo assays for the downstream effects on brain development in mammals. We use exposure schedules and formats resembling those used in OECD test guidelines (TG) for developmental neurotoxicity studies and the Extended One-Generation Reproductive Toxicity Study (EOGRTS; OECD TG 426, TG 443) in rodents. We explore the utility of various methods for the characterisation of brain morphology. We are specifically evaluating the formation of heterotopias, misplaced neurons in the corpus callosum that occur after severe TH deficiency during development. This lesion is the result of the disrupted migration of neuronal cells to the brain cortex. The team around Mary Gilbert at the United States Environmental Protection Agency (US EPA) [11] discovered this form of brain damage after exposing rats to inhibitors of thyroid peroxidase, a key enzyme in the synthesis of THs. We have successfully adopted this method and are ready to test chemicals for their ability to produce this damage to the developing brain (Figure 4).

We are investigating whether similar lesions also occur in the mouse. This will enable us to pinpoint features of the TH system that will impact on species–species sensitivity differences and will help us to assess the human relevance of the rodent models. We also focus on disruptions of cell differentiation and migration, as they materialise in terms of changes in gene expression, cerebellar histogenesis, the altered ratio of neurons vs. glial cells and misplaced neurons left behind during disrupted cell migration. We further evaluate serum TH levels (TSH, T4, T3) and effects on the liver (the main TH metabolising organ) by analysing hepatic gene expression. Ex vivo analyses of the thyroid will enable us to establish several functional readouts, including gene expression profiles, iodine content, deiodinase and dehalogenase activities, based on the non-radioactive Sandell–Kolthoff assay platforms. In this way, we hope to integrate the possible interference of test chemicals at the sites of thyroid hormone synthesis (the thyroid), metabolism and elimination (the liver) and a primary target of TH action during development (the brain).

By robotising the Xenopus Eleutheroembryonic Thyroid Assay (XETA), we build on the existing XETA assay, which has been validated in international ring tests [12]. We are scaling up this test method to enable high throughput testing by robotisation and to develop additional endpoints to make the XETA experiments more informative for assessing the mode of action of the substances. The test relies on transgenic embryos bearing a genetic construct, where Green Fluorescent Protein expression is controlled by THs. In the transgenic embryos, the level of fluorescence reflects the activity of the TH system. The XETA protocol in its present form has been published as an OECD test guideline (TG) 248 [13]. Robotisation will allow us to decrease the cost of each test and to increase the rate of substances screened to medium throughput.

We will evaluate all new test methods in terms of OECD principles of test method validation and in terms of entry into the European Centre for the Validation of Alternative Methods (ECVAM) modular process for the submission of test methods for validation. We will analyse the reliability of the methods (reproducibility of test results within and among laboratories) and their relevance (the relationship between test outcomes and the effects of a chemical in a target species). This work follows the Solna Principles, which require a rationale for the test method, establishing a relationship between the endpoints of a test method and the toxic effect of interest, a formally detailed test protocol (Standard Operating Procedure), the demonstration of intra-test variability, repeatability and reproducibility, the demonstration of a test method’s performance by using reference chemicals (positive controls) and assessments of the new method in relation to existing tests.

#### 2.3.3. Domain 3: Thyroid Hormone System Disruptor Testing in a Multi-Factorial Context and the Development of Comprehensive Testing Strategies

The impact of new test methods developed in domain 2 of the ATHENA project would be rather limited if we did not account for multiple intervening factors in the real world that can either exacerbate or mitigate the effects of chemicals on the TH system. It is also necessary to maximise the impact of new test methods by integrating them into a comprehensive testing strategy for the protection of the developing brain from TH system disruptors. This testing strategy should also support regulatory decision making. The work in domain 3 aims to elaborate on these issues.

We will examine factors that can exacerbate or mitigate the effects of TH system-disrupting chemicals on foetal brain development. A prominent influence with a potentially significant impact is iodine deficiency. This is important to understand, as most testing for TH system disruption is conducted in iodine-rich conditions that are of limited relevance to the iodine-deficient environments often encountered in human populations. The inhibition of iodotyrosine dehalogenase (IYD) will be of particular interest, as this enzyme has the capacity to recycle iodide from side products of TH synthesis. We will also analyse how chemicals that interfere with other hormone systems can modulate the sensitivity of test organisms to TH system disruptors. Considering that thyroid hypofunction is especially prevalent in women, the estrogen sex steroid system deserves particular attention.

We will develop testing strategies based on Adverse Outcome Pathways (AOP) for TH system disruption. This effort will combine existing test methods with the novel methods developed in ATHENA and integrate these into a coherent testing strategy. Our approach will define a sequence of test methods to be used for the evaluation of chemicals for their TH system-disrupting properties and will elaborate upon decision points that specify further testing needs, depending on the outcome of entry point tests. To put the strategy on a sound footing, we will link it with ongoing AOP development activities at the OECD level and extend this to devise a network of quantitative AOP for the TH system. This will enable us to define which elements of the TH system, when disrupted, will have the greatest impact on TH insufficiency or hyperfunction and therefore should be targeted through effective testing. We will develop a workflow that is as parsimonious as possible in terms of resource use, but at the same time extensive enough so that we can reliably identify thyroid disruptors. We will align the testing strategy with regulatory needs for the identification of TH system disruptors, as specified by EU criteria for endocrine disruptors. It is important to define the level of evidence needed to designate a chemical as a TH system disruptor and establish conditions when further testing can be waived. We will demonstrate the functionality and practical applicability of the strategy by examination of a test set of known active and inactive chemicals.

#### 2.3.4. Enhanced International Collaboration, Development of International Strategies for TH System Disruptor Identification and Regulation

To further enhance the impact of our test method developments, we will progress concepts for enhanced international collaboration and development in the area of TH system disruptor identification and regulation.

We will compare international approaches for the regulatory screening, testing and assessment of substances for thyroid-disrupting properties within the EU and among relevant international trading partners, including the USA, Canada, China, Japan, South Africa and Australia. We will first update an existing inventory of completed and ongoing activities related to the screening, priority setting, testing and assessment of chemicals for their endocrine-disrupting properties [1], in as far as this is relevant to thyroid disruptors. A previous deep analysis of differences and commonalities in screening, testing and regulation for TH system disruptors in different EU and non-EU legalities [14] has shown considerable inconsistencies in terms of toxicity values and assessment factors used for endocrine disruptor standard setting. Building on this analysis, we will map out a strategy for international harmonisation that considers the diversity of protection goals enshrined in the body of law of EU and non-EU legalities.

## 3. Discussion

The deficiencies of current test guidelines in the OECD Conceptual Framework for endocrine disruptors are well recognised. The absence of sensitive parameters for the detection of downstream adverse effects diagnostic of TH system disruption on the developing brain is a major gap in these guidelines and is likely to hamper the reliable detection of chemicals as endocrine disruptors. Detailed suggestions for improving test guidelines have been made, especially regarding the inclusion of endpoints for the identification of adverse effects on brain development and brain morphology [1].

The ATHENA project responds to these suggestions and recommendations and it is hoped that the ATHENA partnership will achieve significant improvements upon completion of the project.

Three EU chemical regulations are relevant to chemicals that can disrupt the TH system: Commission Regulation (EU) 2018/605136, an amendment of the Plant Protection Products Regulation and Commission, and Delegated Regulation (EU) 2017/210072 which addresses the Biocidal Products Regulation (EU) No 528/201252. Both these regulations define scientific criteria for the determination of the endocrine-disrupting properties of plant protection products and biocides, respectively. According to these criteria, endocrine disruptors are substances that:

- Show adverse effects in an intact organism or its progeny, which is a change in the morphology, physiology, growth, development, reproduction or life span of an organism, system or (sub)population that results in an impairment of functional capacity, an impairment of the capacity to compensate for additional stress or an increase in susceptibility to other influences;
- Have an endocrine mode of action (MOA), i.e., alter the function(s) of the endocrine system;
- Exhibit adverse effects because of an endocrine MOA.

The third relevant regulation, the European Regulation REACH (EC 1907/2006), defines endocrine disruptors, including TH system disruptors, as substances of very high concern (SVHC) if they induce effects on human health that give rise to a level of concern similar to those of carcinogens, mutagens and reproductive toxicants (CMR) or persistent, bioaccumulative or toxic (PBT) or very persistent and very bioaccumulative chemicals (vPvB).

The challenge for ATHENA is to operationalise the scientific criteria for identifying endocrine disruptors into test outcomes relevant to TH system disruption. This will take time. In the interim, it is necessary to make regulatory decisions based on the inadequate data. The European Food Safety Authority (EFSA) and the European Chemicals Agency (ECHA) have developed guidance for the application of the endocrine disruptor criteria based on the outcomes of currently available tests [15]. This guidance document proposes the following approaches for the interpretation of data from animal studies in the context of identifying chemicals that disrupt the TH system:

- Substances inducing histopathological changes in the thyroid (focal hyperplasia and/or neoplasia), with or without changes in the circulating levels of TH, are regarded as posing a hazard for human TH insufficiency in adults, as well as the pre- and post-natal neurological development of offspring;
- Substances that alter circulating levels of T3 and/or T4 without histopathological findings are also seen as presenting a potential concern for neurodevelopment;
- In the absence of substance-specific data which provide proof of the contrary, humans and rodents are considered equally sensitive to TH system disruption (including cases where liver enzyme induction is responsible for increased TH clearance).

This guidance reflects the uncertainty about how TH system disruption can be appropriately identified in the face of a lack of relevant testing methods. Upon completion of the ATHENA project, we will be able to place the identification of TH system disruptors on a sound footing.

## Abbreviations

AOP	Adverse Outcome Pathway
DIO	Deiodinase
ECHA	European Chemicals Agency
ECVAM	European Centre for the Validation of Alternative Methods
EFSA	European Food Safety Authority
EOGRTS	Extended One-Generation Reproductive Toxicity Study
HTS	High Throughput Screening
EROD	7-Ethoxyresorufin O-dealkylase
IYD	Iodotyrosine dehalogenase 1
MCT8	Monocarboxylate transporter 8
MOA	Mode of Action
NIS	Sodium iodide symporter
OECD	Organisation for Economic Cooperation and Development
PROD	Pentoxyresorufin O-dealkylase
QSAR	Quantitative Structure–Activity Relationship
T3	Triiodothyronine
T4	Thyroxine
TBG	Thyroxin-Binding Globulin
TG	Test Guideline
TR	Thyroid Receptor
TRH	Thyrotropin-Releasing Hormone
TSH	Thyroid Stimulating Hormone
TSHR	Thyroid Stimulating Hormone Receptor
TTR	Transthyretin
UDPGT	Uridine 5′diphospho-glucuronosyltransferase
